# Zip nucleic acid: a new reliable method to increase the melting temperature of real-time PCR probes

**DOI:** 10.1186/2251-6581-13-26

**Published:** 2014-02-04

**Authors:** Ehsan Alvandi, Fariba Koohdani

**Affiliations:** 1 Diabetes Research Center, Endocrinology and Metabolism Clinical Sciences Institute, Tehran University of Medical Sciences, Tehran, Iran; 2 Department of Cellular Molecular Nutrition, School of Nutritional Sciences and Dietetics, Tehran University of Medical Sciences, Tehran, Iran

**Keywords:** ZNA, Zip nucleic acid, Melting temperature, MGB, Minor groove binder, Probe, SNP genotyping, Real-time PCR

## Abstract

TaqMan genotyping with real-time PCR is a reliable method for single nucleotide polymorphism detection, which is done by probes. These oligonucleotides should be short enough to avoid mismatch hybridization, as well as having 5–10°C higher melting temperature than the primers of real-time PCR reaction. One approach for these qualities is to conjugate the probe with minor groove binder (MGB). Having no access to MGB probes, we searched for an alternative. In the current study, we used Zip Nucleic Acids (ZNA) as probes to increase its stability and melting temperature. Our aim was to genotype the -265 T/C changes of Apolipoprotein A-2 gene. We set up the real-time PCR reaction with ZNA probes, and by repeating the reactions, we confirmed the reliability of this new approach. It is now recommended to use ZNA probes, as an alternative to MGB probes, to increase the probe Tm value and its binding to target DNA.

## Background

A single-nucleotide polymorphism (SNP) is the variation of a single nucleotide in DNA sequence between two or more alleles. SNP genotyping is the study of such variations across the genome. It can be applied with different methods. Among them, TaqMan genotyping assay with the real-time PCR is a gold standard for the analysis of a limited number of SNPs in large sample collections, specifically when the site of an SNP is not detectable with a restriction enzyme. It allows the detection of both SNP alleles in a single reaction tube [1,2].

In our study, the desired SNP was not detectable with any restriction enzyme. This SNP is in the promoter region of Apolipoprotein A-2 gene (−265 T/C, rs5082). Therefore, we decided to carry out the TaqMan genotyping assay with real-time PCR. In order to have the most reliable result, we had to detect both alleles simultaneously. Thus we applied two different probes in each reaction. The aim of the study was to analyze this SNP among 800 Diabetes mellitus type 2 patients.

## Main text

Technically, in TaqMan genotyping, each probe is a linear oligonucleotide with a fluorogenic dye attached to the 5′ end and a quencher molecule to the 3′ end. The close proximity of the reporter and quencher prevents the fluorescence emission. In each PCR cycle, after primer and probe annealing, the Taq polymerase attaches to the 3′ end of the primer and synthesize the new complementary strand. The Taq polymerase used in TaqMan genotyping has an additional 5′ to 3′ exonuclease activity. This causes the degradation of probe, and thus the release of the reporter dye fluorescence [2,3].

The point is the annealing temperature of probes and primers. These oligonucleotides should be designed in a way that in reaction, first the probes, and then the primers should be hybridized with the DNA. So the melting temperature (Tm) of probes should be more than primers. Simply, it means the probes should be longer than primers. However, longer the probes increase the chance of mismatch formation between probes and target DNA. Longer probes are less sensitive to mismatch discrimination. This will cause false positive results, especially when the difference between 2 or more probes is only a single nucleotide. Therefore, designing longer probes is not the right solution [3].

In order to increase the Tm value of the probes, without increasing the probe length, one way is to use MGB (Minor Groove Binder). Attaching MGB to the 3′ end of TaqMan probes increases the stability of hybridization between the probe and target DNA. This results in the increase of probe melting temperature. By increasing the Tm value, the design of shorter probes is now possible. Shorter probes make them more specific to determine a single nucleotide difference. By using the shorter probes with higher Tm values, there would be no risk of mismatch formation between a false probe and the target DNA molecule [3-5].

In Iran, we do not have access to TaqMan probes, since they are only produced by Applied Biosystems, which is an American company. We had to look for an alternative way to increase the Tm value of probes. We searched many companies which deals with oligonucleotide synthesis, and finally found that a German company, Metabion Inc., has just introduced a new method to increase the Tm value, called ZNA.

Zip nucleic acid (ZNA) is generally an oligonucleotide which is conjugated with a cationic compound, named spermine. The role of spermine is to increase the affinity between the oligonucleotide and the target nucleic acid molecule. This results in decreasing the electrostatic repulsion between these two negatively charged molecules [6]. The melting temperature of the oligonucleotide will increase according to the number of conjugated spermine in a linear manner. It can raise the Tm of 8-12-mer oligonucleotides up to 70°C. Furthermore, ZNAs retain the ability to discriminate between a perfect match and a single base-pair-mismatched complementary sequence, thus increasing the accuracy of detection [7]. Recent studies confirm the reliability of Zip nucleic acids, either as primers [8] or real-time PCR probes [9].

In our study, we used the ZNA probes to genotype the -265 T/C changes of Apo A-2 gene. We made use of ZNA unique features for increasing the Tm value of our probes, and thus to avoid having a mismatch between the false probe and the target DNA. The probe sequences are: ApoA2-Ca: 5′-FAM- TTGGACTTGAGTGCAACA-BHQ1-3′; and ApoA2-Ta: 5′-JOE- CTTGGACTTGAATGCAACA-BHQ1-3′. The specific nucleotide for detection of T > C change is underlined. Without the spermine, the Tm of probes is 47.5°C and 49.8°C respectively; calculated by Nearest Neighbor method [10]. By using ZNA probes, the Tm value raised to 67°C. This ensures the reliability of the results.

PCR was performed in a 10 μL final volume. The reaction mixture contained 5 μL TaqMan Universal Master Mix II (Applied Biosystems), 200 nmol/L of both probes, 900 nmol/L of both forward and reverse primers (all from Metabion AG), and 25 ng genomic DNA. The thermal cycler was run with 40 cycles at 95°C for 15 s and 60°C for 1 minute. Allelic discrimination was performed at the end of each cycle and on the post-PCR product. The reaction was run by StepOne Real-Time PCR System (Applied Biosystems).

**Figure 1:**
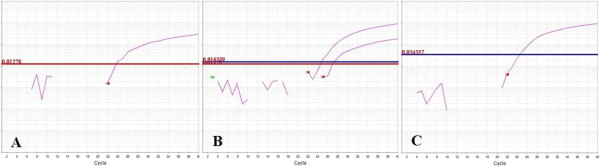
**Amplification plot data of three samples.** The data gathered through the reaction at the end of each PCR cycle. The red and blue lines indicate the base line of T and C alleles respectively. The number above each base line indicates the delta Rn value. **A)** TT homozygote; **B)** TC heterozygote; **C)** CC homozygote.

**Figure 2:**
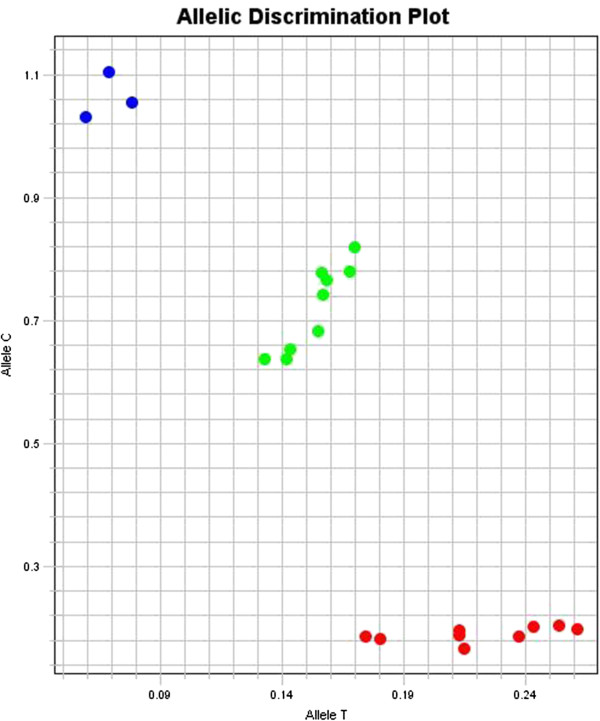
**Final result of a real-time PCR run for 24 samples, which is collected at post-PCR stage.** Red, green and blue dots represent TT homozygote, TC heterozygote and CC homozygote samples, respectively.

Before studying patient samples, we repeat the test on our reference DNA samples in triple times, whether in the same or different runs to ensure the reliability of our PCR setup. Moreover, we randomly chose some patient samples DNA and repeat the test on them. All this repetitions lead to the same results. Figure 1 shows the amplification plots of three allelic combinations, and Figure 2 shows the final result of a real-time PCR run.

## Discussion

Zip Nucleic Acids are new in the field of oligonucleotide synthesis. Recently, two new researches are reported, which have used this type of oligonucleotides. Moreau et al. used ZNA as primers. They reported that these primers can improve the yield of cDNA synthesis, especially for the genes expressed at low levels [8]. In the second study, Paris et al. compared ZNA probes with MGB probes. They have proved that both of them behaved similarly. They show that under standard PCR conditions, ZNA probes can exhibit high performances [9].

In the current study, we also set up our experiment with ZNA probes. We run real-time PCR of 800 samples, and we repeat some of these tests. In all cases, we reached the same results. Therefore, we confirmed that this type of oligonuclotide is reliable to be used as real-time PCR probe. This study is another proof to show the accuracy of Zip Nucleic Acids.

## Conclusion

While having no access to MGB-conjugated TaqMan probe, we decided to use an alternative, called ZNA to increase the melting temperature of the probes. Our data suggest that these probes can provide an effective and reliable alternative to MGB for SNP genotyping.
